# Editorial: Translational research in severe COVID-19 and long-term symptoms post-COVID-19

**DOI:** 10.3389/fmed.2023.1261211

**Published:** 2023-09-27

**Authors:** Vanesa Vicens-Zygmunt, Gloria Pérez-Rubio, Leslie Chavez-Galan, Ivette Buendia-Roldan, Ramcés Falfán-Valencia

**Affiliations:** ^1^Interstitial Lung Disease Unit, Department of Respiratory, University Hospital of Bellvitge, CIBERES, University of Barcelona (UB)-IDIBELL, Barcelona, Spain; ^2^HLA Laboratory, Instituto Nacional de Enfermedades Respiratorias Ismael Cosio Villegas, Mexico City, Mexico; ^3^Laboratory of Integrative Immunology, Instituto Nacional de Enfermedades Respiratorias Ismael Cosio Villegas, Mexico City, Mexico; ^4^Translational Research Laboratory on Aging and Pulmonary Fibrosis, Instituto Nacional de Enfermedades Respiratorias Ismael Cosio Villegas, Mexico City, Mexico; ^5^HLA Laboratory, Instituto Nacional de Enfermedades Respiratorias Ismael Cosio Villegas, Mexico City, Mexico

**Keywords:** COVID-19, long COVID-19, post-COVID-19, post-COVID-19 syndrome, SARS-CoV-2

In addition to acute and severe symptoms of SARS-CoV-2 infection, many patients worldwide suffer persistent symptoms from post-COVID-19 syndrome (PCS).

This Research Topic aims to publish original translational research and review articles contributing to developing additional knowledge of PCS, considering the acute infection that contributes to developing post-COVID symptoms and mortality and thus trying to identify target markers for managing the affected subjects.

In this Editorial, we have summarized 13 manuscripts: 5 related to acute infection, seven concerning the role of molecular and clinical factors of PCS, and 1 *in vitro* research study about preventive therapy.

Acute SARS-CoV-2 infection could severely compromise the subject's health. There is a decreasing trend of deaths, hospitalizations, and intensive care unit (ICU) admissions, principally due to vaccination, acquired post-infection immunity, and less aggressive virus variants.

Concerning mortality, Martin-Conty et al. in a prospective, multicenter, ambulance-based ongoing study in Spain, observed long-term mortality as the primary outcome in acute patients treated by emergency medical services, and COVID-19 was observed as an independent risk factor for long-term mortality. They identified that patients who previously experienced an acute COVID-19 episode presented a mortality rate almost twice that of non-COVID-19 subjects, suggesting, with a final model adjustment, that COVID-19 was a risk factor for long-term mortality.

Due to the elevated mortality in hospitalized COVID-19 patients, there is an increased interest in finding serum biomarkers predicting mortality to adopt beneficial measures and easy protocols during the post-COVID follow-up. From Mexico, Cortes-Tellez et al. analyzed serum levels of different parameters from a routine laboratory in a cohort of severe COVID-19 hospitalized patients. They observed in a multivariate analysis that leukocytes and neutrophils were the best biomarkers for predicting mortality risk independently of age, gender, or comorbidities. The authors concluded the importance of using them routinely.

Likewise, Pavan Kumar et al. in a comparative research study from India, elucidated the role of matrix metalloproteinases (MMPs) in the pathogenesis of pediatric COVID-19, examining the MMPs plasma levels in children with Multisystem Inflammatory Syndrome (MIS-C) and acute COVID-19 and comparing them to convalescent COVID-19 and children with other common tropical diseases. Higher levels of MMPs were observed in children needing ICU admission. Lastly, MMP levels showed a significant correlation with laboratory parameters, comprising CRP, ferritin, lymphocytes, D-dimer, and sodium levels, and the authors proposed that MMPs play a crucial role in the MIS-C and COVID-19 pathogenesis in children and may help distinguish MIS-C from other conditions with an overlapping clinical phenotype.

On the other hand, SARS-CoV-2 reinfection is also a topic because it impacts sequels and illness severity. Suljic et al. from Slovenia, showed in an observational case-control study that reinfections with the Delta variant generate fewer hospitalizations than first infection, suggesting the development of more robust immunity protection developed by infected individuals and also vaccinated individuals (hybrid immunity). This study provides additional insight into reinfection, which may allow appropriate public health measures to be taken.

Concerning the imaging study of COVID-19 pneumonia evolution during hospitalization, lung ultrasound (LUS) has been extensively used during the COVID-19 pandemic. Blair et al. from the United States, prospectively studied 244 moderate (non-ICU) and severe (ICU) COVID-19 hospitalized adults in a longitudinal cohort to evaluate the association between LUS characteristics and clinical severity. The authors described that at baseline, B-lines (edema, fibrosis, inflammation) were more prevalent in severe patients than in moderate ones. However, no significant differences were found between severe and moderate illness over time. Thus, the authors do not support the use of serial LUS to monitor the progression of disease severity.

Pulmonary fibrosis due to SARS-CoV-2 infection is a significant concern (1). A study performed on postmortem patients in Spain (Pérez-Mies et al.) documented the evolution of diffuse alveolar damage (DAD) to the fibrosing pattern and defined the transcriptional programs involved. The authors analyzed lung autopsy samples from five lobes of 33 patients with a severe and prolonged SARS-CoV-2 course. They found that progression to fibrosis in severe COVID-19 was associated with overexpression of fibrogenic pathways (PI3K-AKT) and significant expression of SPARC and CTHRC1 in exudative-fibrosing DAD compared with the control. Whereas downregulation of the Hippo pathway was observed (suggesting epithelial cell damage response), the authors did not observe any role in the epithelial–mesenchymal transition in the fibrosis process. They suggested a possible role of viral persistence in maintaining lung damage.

Concerning PCS, we know that at least 65 million individuals around the world are suffering from this multisystemic condition comprising persistent and severe symptoms lasting at least 2 months, usually after 3 months of acute SARS-CoV-2 infection that is not explained by another diagnosis (2). Different terms such as long COVID, persistent post-COVID, and post-acute COVID-19 syndrome define the same condition. Several hypotheses have been proposed to explain this syndrome. Predicting which patients will develop PCS is a challenge. In this sense, Lai et al. from Massachusetts, addressed an interesting systematic review to determine potential prognostic serum biomarkers for long COVID. They concluded that the persistence of up-regulation of IL-6, CRP, and TNF-α might present potential diagnostic biomarkers of PCS. In patients with neurological symptoms, neurofilament light chain (NFL) and glial fibrillary acidic protein (GFAP) in serum may serve as diagnostic biomarkers, and the authors proposed to evaluate IL-4, IFN-α, CCL2, ferritin, and hemoglobin too. They also suggested evaluating CXCL10, TGF-β, IFN-β, and IL-1α in patients with pulmonary symptoms.

Another interesting topic in the follow-up is the sequelae in computed tomography (CT) and their association with risk factors. Rincon-Alvarez et al. reported in their Colombian cohort that older age, male sex, and ICU admission were related to typical patterns of admission CT and that a third of patients with moderate and severe COVID had abnormal lung computed tomography at 6-month follow-up.

Concerning health-related quality of life (HRQoL) in patients with PCS, Ahmad et al. employing a multicenter cohort study in a Swedish population, explored the frequency of self-reported continued symptoms and diminished HRQoL in relation to functional exercise capacity 6 months after infection, and they also explored risk factors for COVID-19 sequelae. Hospitalization was a significant risk factor for developing persistent symptoms, reduced overall health, and post-acute COVID syndrome (PACS). They concluded that persistent symptoms and reduced HRQoL are frequent in COVID-19 survivors and that patients requiring hospitalization due to severe infection were more likely to develop PACS.

Furthermore, Al-Husinat et al. looking for the prevalence of PCS after mild-to-moderate COVID-19 in the Jordanian population, applied the Newcastle PCS Follow-up Screening Questionnaire and found that mood disturbance followed by fatigue, anxiety, and myalgia were the most frequent PCS symptoms. Female sex substantially raised the risk for multiple PCS symptoms. They concluded that PCS is highly prevalent among COVID-19 survivors, especially in female patients and patients with comorbidities, and also recommended physical and mental rehabilitation.

In contrast, Román-Montes et al. analyzed the prevalence, symptoms, and HRQoL of PCS in a retrospective cross-sectional study of 246 Mexican patients who required hospitalization because of severe infection. They determined a prevalence of 76% of PCS in patients with a median age of 55 years. It was associated with smoking, severe COVID-19, lower arterial blood oxygen saturation on admission, extensive lung involvement, and elevated fibrinogen levels. Moreover, the most frequent symptoms of PCS were difficulty concentrating (81%), dyspnea (75%), and arthralgia (71%). They suggested identifying diagnostic and therapeutic interventions to restore health and QoL in those patients.

However, no successful treatment is currently offered for managing PCS symptoms, while only rehabilitation programs are promoted, and regular drugs are prescribed for supportive therapies (3). Concerning rehabilitation programs in PCS, Allendes et al. from Chile performed a systematic review on cardiovascular and autonomic dysfunction in PCS. They concluded that alterations in the autonomic nervous system partially mediate cardiovascular sequelae of COVID-19 infection. They hypothesized that applying new cardiovascular rehabilitation programs should allow healthcare personnel to manage the consequences of long-term COVID-19.

Dissook et al. reported on a study testing the activity of phytochemical polyphenol compounds (rosmarinic acid and luteolin) from Perilla frutescens in an *in vitro* lung cell model of SARS-CoV-2-induced inflammation. They documented that these compounds inhibited SARS-CoV-2 spike S1-induced inflammatory responses in A549 cells in a dose-dependent manner, seemingly through the JAK1/STAT3-NLRP3 inflammasome axis, at both the gene transcription and protein levels. They concluded that luteolin and P. frutescens may be potential candidates in the preventive therapeutic strategy for inflammation-related post-acute sequelae of COVID-19.

The present Research Topic contributes novel information toward a better understanding of the possible biomarkers and risk factors contributing to post-COVID symptoms, mortality, radiologic and histologic evolution, and potential preventive therapeutic plants and rehabilitation programs to improve the QoL of PCS patients.

## Author contributions

VV-Z: Investigation, Methodology, Writing—original draft, Writing—review and editing. GP-R: Conceptualization, Investigation, Methodology, Validation, Writing—original draft, Writing—review and editing. LC-G: Investigation, Methodology, Writing—original draft, Writing—review and editing. IB-R: Conceptualization, Investigation, Methodology, Writing—original draft, Writing—review and editing. RF-V: Conceptualization, Investigation, Methodology, Project administration, Resources, Supervision, Validation, Visualization, Writing—original draft, Writing—review and editing.
